# Recording and quantifying sympathetic outflow to muscle and skin in humans: methods, caveats and challenges

**DOI:** 10.1007/s10286-020-00700-6

**Published:** 2020-06-25

**Authors:** Vaughan G. Macefield

**Affiliations:** 1 Human Autonomic Neurophysiology Laboratory, Baker Heart and Diabetes Institute, 75 Commercial Rd, Melbourne, VIC 3004 Australia; 2 Department of Physiology, University of Melbourne, Melbourne, VIC Australia

**Keywords:** Muscle sympathetic nerve activity, Skin sympathetic nerve activity, Multi-unit recording, Single-unit recording, Microelectrodes, Microneurography

## Abstract

The development of microneurography, in which the electrical activity of axons can be recorded via an intrafascicular microelectrode inserted through the skin into a peripheral nerve in awake human participants, has contributed a great deal to our understanding of sensorimotor control and the control of sympathetic outflow to muscle and skin. This review summarises the different approaches to recording muscle sympathetic nerve activity (MSNA) and skin sympathetic nerve activity (SSNA), together with discussion on the issues that determine the quality of a recording. Various analytical approaches are also described, with a primary emphasis on those developed by the author, aimed at maximizing the information content from recordings of postganglionic sympathetic nerve activity in awake humans.

## Introduction

Microneurography, in which an insulated tungsten microelectrode is inserted through the skin into an accessible peripheral or cranial nerve, allows one to record the electrical impulses (action potentials) generated by myelinated and unmyelinated axons of either afferent (sensory) or efferent (motor) types. This review provides a brief history of the development of microneurography and considers the different means by which sympathetic nerve activity—as recorded directly from postganglionic sympathetic axons in awake human participants—can be analysed. There have been several reviews on the history of microneurography [1–4], and several recent reviews that consider the technical aspects of sympathetic nerve recordings [5–9], to which the reader is directed. The aim of the current review is to present some different methods of analysing sympathetic outflow in human health and disease. In many respects it is also a personal narrative, documenting my 33 years of experience with microneurography, yet I acknowledge the collective wisdom of the many investigators around the world using this technique.

Developed by Karl-Erik Hagbarth and Åke Vallbo at the Academic Hospital in Uppsala, Sweden, in the early 1960s, these investigators repeatedly impaled each other’s nerves (including the ulnar, radial, tibial and common peroneal nerves) to develop the optimal electrode and insulation material, settling on tungsten with an epoxy coating. Given their interest in muscle spindles, and having recorded from these in the cat, their initial aim was to attempt to record multi-unit activity from human muscle spindle afferents to address the popular servo-control theory of motor control [10]. Their success in obtaining multi-unit recordings was soon overtaken by the realisation that they were able to record action potentials generated by individual myelinated afferent axons, both those supplying muscle spindle endings [11] but also cutaneous mechanoreceptors [12]. This discovery led to decades of ground-breaking studies in human somatosensory physiology and sensorimotor control, including the early observation that muscle spindles were recruited or increased their discharge *after* the onset of a voluntary contraction, not before [13], and documenting the firing properties of the separate classes of low-threshold mechanoreceptors in the skin [14, 15]. But another surprise awaited them, as documented in comprehensive reviews on the development of microneurography by these authors [1–3]. While initially considered to be electrical artifacts, Hagbarth and Vallbo soon realised that the slow spontaneous bursts of activity they observed in their recordings represented the mass activity of sympathetic axons, i.e. the unmyelinated postganglionic sympathetic fibres directed to effector organs in muscle or skin [16, 17]. This led to the development of sympathetic microneurography by Gunnar Wallin in Uppsala, and the training of many investigators who travelled to Uppsala and returned to their laboratories around the world. Independently, Tadaki Mano later developed sympathetic nerve recordings in Nagoya, and went on to train many researchers in Japan, such that they have their own Microneurography Society. Direct recordings of muscle sympathetic nerve activity (MSNA) and skin sympathetic nerve activity (SSNA) are now made from many laboratories throughout the world, representing the bulk of studies employing microneurography. Such recordings have contributed much to our understanding of the operation of the sympathetic nervous system, both in healthy individuals and those with cardiovascular, respiratory, renal or neurological diseases. However, because of the time taken to obtain a suitable intraneural recording it is unsuitable for routine diagnostic purposes. Moreover, the high variability in resting sympathetic outflow to muscle amongst healthy participants made it difficult to establish a clear baseline to which pathophysiological changes could be referenced. Nevertheless, investigational studies in well-defined patient groups have contributed much to our understanding of the pathophysiological changes in sympathetic outflow in different disease states. Importantly, while the technique is invasive, when performed by trained investigators it is considered a safe procedure. Of course, ethics approvals must be in place before undertaking microneurography and universal precautions must be adhered to. Moreover, all research is conducted on volunteers and good communication between the investigator and the participant is essential; as we will see below, any sensations reported by the participant provide very useful clues to the microneurographer, who should also prompt the participant to report any sensations—painful or otherwise. In my experience, the vast majority of participants are willing to return for another recording session and do not report any untoward effects. A generally accepted standard is not to enter the same nerve until 4 weeks following the first recording session. A recent meta-analysis has confirmed that long-term sequelae are indeed very rare [18].

## Recording sympathetic nerve activity

Before we consider analytical methods, it is worth going over the recording approach. Sympathetic nerve activity is usually recorded from the common peroneal nerve (also known as the fibular nerve) as it courses behind the fibular head, though the Japanese favour the tibial nerve, which they enter through the popliteal fossa. Interestingly, they use a much longer and more flexible tungsten microelectrode than the standard microneurography needle, which has a shaft diameter of 0.2 mm, and use forceps to insert the microelectrode through the skin. This nerve is composed of fascicles that supply the triceps surae muscles and the intrinsic muscles of the foot and cutaneous fascicles that supply the plantar and lateral aspects of the foot. For most other laboratories, the common peroneal nerve is chosen because, with one exception, at the level of the fibular head the nerve is composed of distinct fascicles supplying either the pretibial flexors (tibialis anterior, the toe extensor muscles or the peronei muscles) or skin on the dorsum of the foot or anterolateral aspects of the leg (and an occasional fascicle on the medial aspect of the foot). The exception is extensor hallucis longus (EHL): while this fascicle supplies motor and sensory axons to the muscle that dorsiflexes the big toe, it also supplies a small patch of skin between the first and second toes. This fact makes this particular fascicle problematic, given that sympathetic nerve activity recorded from this fascicle can be composed of bursts of both MSNA and SSNA, leading to ambiguous findings. So, knowing the identity of the fascicle is important: is it supplying muscle, or skin or both? If both, how can one interpret the sympathetic bursts? Other nerves, including the median and ulnar nerves in the upper arm are even more mixed [19] as there is less fascicular organization in proximal sections of limb nerves, with cutaneous and muscle regions of the nerve showing admixture within the nerve. Although it may be more difficult to find, the radial nerve in the upper arm does offer certain advantages: it is composed of distinct muscle or cutaneous fascicles, and allows one to record MSNA during lower-limb exercise, for example. As for more distal segments, for the median and ulnar nerves at the wrist there is a distinct organization of fascicles into those supplying muscle or skin, yet it is surprisingly difficult, though not impossible, to encounter spontaneous bursts of sympathetic nerve activity within fascicles of these nerves.

For me, the gold-standard approach is to use the original Swedish methodology (which I learnt initially from David Burke—who brought the technique to Australia—and then from Karl-Erik Hagbarth, Åke Vallbo, Gunnar Wallin and Roland Johannson), in which electrical stimuli are delivered through the microelectrode to assist in guiding the microelectrode tip into a fascicle of the nerve. First, a 1–2 mm external probe (with a dab of conductive gel on its tip) is used to deliver brief cathodal (negative) pulses through the skin (0.2 ms, 1–10 mA, 1 Hz) from an electrically isolated stimulator certified for human connection; the anode (a Ag/AgCl surface electrode) is attached to the opposite side of the knee so that current passes directly through the limb. The aim here is to track the course of the nerve; the sites requiring the lowest current to evoke a muscle twitch and/or radiating paraesthesiae represent the locations at which the nerve is closest to the skin. After one has identified the optimal site(s), an insulated tungsten microelectrode is inserted a few millimetres through the skin, and then weak electrical pulses (0.2 ms, 0.01–1.0 mA, 1 Hz) are delivered through the microelectrode. Note that the currents delivered through the tip of the microelectrode are an order of magnitude smaller than those delivered through an external probe; higher currents are required for external stimulation because of the high impedance of the skin. The rationale here is that the electrical pulses will identify the nerve, if the electrode tip is close enough, by exciting myelinated motor and cutaneous axons. To get to this point the stimulus intensity is slowly increased until a maximum of 1 mA, or until a twitch is observed or cutaneous sensations reported. The experimenter then manually advances the microelectrode, noting the effects of slight changes in angle of the microelectrode shaft on the evoked responses: by carefully adjusting the angle and depth of the microelectrode one can penetrate a fascicle of the nerve. This typically occurs at stimulus currents of 0.02 mA or less, at which current the identity of the fascicle is known from the actions of a given muscle or sensations reported in the fascicular innervation zone on the skin. A variant of this approach, developed by Åke Vallbo when he moved from Uppsala to take up a faculty position at the University of Umeå, involves inserting an uninsulated guide microelectrode through the skin and delivering weak pulses through this electrode. Once the nerve is found, an insulated microelectrode of identical length is then inserted through the skin in parallel to the guide needle, and the nerve impaled by reaching the same depth and angle as the guide needle. The development of this approach was to prevent the electrical stimuli from damaging the insulation, though this has been shown not to seriously affect the impedance of the microelectrodes. Rather, it is passing through the different tissue layers, especially the tough nerve sheath and fascicular walls, that removes some insulation and hence lowers its impedance; this makes the microelectrode less focal in its capacity to record, with single-unit recordings typically requiring microelectrode impedances greater than 300 kΩ.

**Fig. 1:**
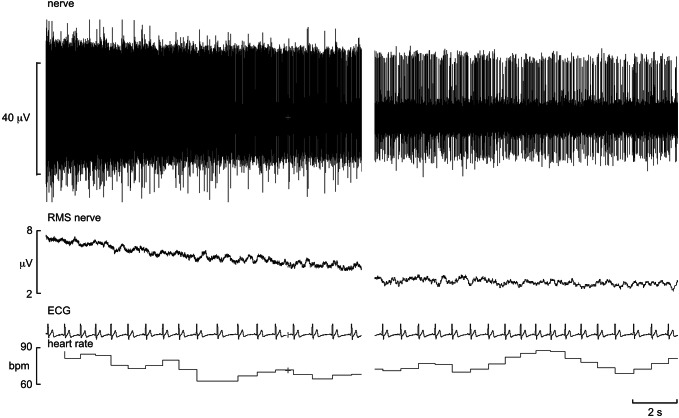
Recording of ectopically generated discharges from a muscle fascicle of the common peroneal nerve on microelectrode entry into the fascicle. The panel on the right is the same recording 8 s later, showing that it can take a while for this “insertion discharge” to settle down. RMS = root mean square (200 ms moving average). Unpublished data from Macefield

Not all investigators use the systematic approach of delivering electrical stimuli through the recording (or guide) microelectrode, however, with some preferring to direct the microelectrode into the nerve by sound. They do this by listening to the amplified signals as the microelectrode tip passes through the tissues surrounding the nerve and the telltale insertion discharges produced by mechanical irritation of axons as the tip penetrates a fascicle. An example is shown in Fig. 1. This recording was captured as the microelectrode tip entered the fascicle supplying the tibialis muscle. It is always wise to wait for this discharge to die down, as one may find that an individual axon has been impaled: in this case, the ectopic discharges eventually settled into the regular discharge of a muscle spindle afferent. While the “blind” approach clearly works, there is a small risk of damaging the tip of the microelectrode if it hits bone. Those who use this approach may do so out of concern about electrically stimulating within a nerve and causing pain in the participant, which is not unreasonable. However, we have never found this to be a problem: healthy participants and clinical patients alike tolerate intraneural stimulation very well, but the process has to be followed carefully. While the microneurographer is adjusting the angle and depth of the microelectrode, an assistant is controlling the delivery of electrical stimuli (0.2 ms pulses, 1 Hz, 0.01–1.0 mA) to the microelectrode. The aim here is to find a threshold current that evokes a muscle twitch or cutaneous sensation, and then to move the microelectrode and determine whether the twitch or sensation becomes stronger or weaker. The communication between the microneurographer and the person controlling the stimulator is key, as is the feedback provided by the participant. This approach allows one to enter a fascicle of the nerve typically within 5–20 min, and the identity of the fascicle is then known when a distinct twitch or sensation is evoked at currents of 0.02 mA or lower. We always inform the participant that they may experience some transient discomfort, lasting less than a second, as the microelectrode is advanced during intraneural stimulation but when they see their muscles twitching they invariably laugh, as they cannot voluntarily control their muscle. Likewise, electrically evoked paraesthesiae are weak and their location can be readily reported by the participant, again guiding the microneurography towards this fascicle for recording SSNA or away from it if the aim is to record MSNA. Others use ultrasound guidance to direct the microelectrode into the nerve, which is particularly helpful in people who are obese: the electrode is highly echogenic, so can be visualized as it is advanced toward the nerve [20]. Nevertheless, while this approach definitely works, one does not know the identity of the fascicle that one has impaled, so the various means of identifying the fascicle reported above need to be carried out.

Regardless of how the microelectrode tip is introduced into a fascicle, it is important to ask participants about any sensations they may feel during the procedure. Microneurography is not intrinsically painful, but it can induce transient discomfort; whether this persists depends on the communication between investigator and participant. This feedback is instructive: a dull ache at the site of the microelectrode insertion is typically associated with the tip of the microelectrode being close to the nerve sheath or fascicular wall (these structures are heavily innervated with nociceptors). Conversely, a deep dull ache within a muscle, which may include an ache referring distally (sometimes proximally) and occasionally a fasciculation in the muscle, suggests that the microelectrode tip has penetrated a muscle fascicle. Conversely, radiating paraesthesiae and/or a shooting pain indicates that the tip has penetrated a cutaneous fascicle. One defines the innervation territory of a cutaneous fascicle by light stroking of the skin, which—as long as the stimuli are not sufficient to induce muscle stretch—does not generate responses when one is recording from a muscle fascicle.

Both the muscle and the cutaneous sensations are produced by the mechanical generation of ectopic impulses in axons at the site of impalement. It is important to ask the participant whether any of these sensations are felt when the microelectrode is first inserted through the skin: it occasionally happens that the microelectrode impales the nerve immediately on insertion in lean participants with a very superficial nerve. When one records these discharges it is notable that a barrage of impulses recorded from a muscle fascicle is rarely perceived, such was the case in Fig. 1, whereas a barrage of impulses in a cutaneous fascicle usually is perceived. This fits with the fact that microstimulation of single muscle spindle afferents does not generate a percept, whereas microstimulation of most tactile afferents does; evidently many muscle spindle afferents need to be excited to generate a meaningful perception of muscle stretch [21].

Because the action potentials generated by unmyelinated (C-fibre) sympathetic axons are so small, they need to be amplified approximately 20,000 times via an electrically isolated headstage located close to the recording site. The wires connecting the headstage to the microelectrodes need to be kept short (no longer that 10 cm) to avoid electrical interference; long leads connected to a high-impedance microelectrode act as antennae and pick up electrical noise. Most commercially available headstages pre-amplify the signal 10–100 times, with further amplification and filtering being undertaken in the main amplifier. Once in a muscle fascicle, muscle stretch or tendon percussion is applied to identify muscle spindle afferents, many of which may be spontaneously active owing to the prevailing degree of stretch in the parent muscle; this is more common for the tibialis anterior muscle—the primary dorsiflexor of the ankle—owing to the weight of the foot stretching the relaxed muscle. The presence of muscle spindle activity confirms that the microelectrode tip is in a defined muscle fascicle, allowing the experimenter to manually guide the microelectrode tip into an area of the fascicle in which spontaneous bursts of MSNA are encountered. Sympathetic nerve activity is typically recorded with a bandpass of 300 Hz to 3 or 5 kHz, though many prefer a narrower bandwidth of 700 Hz to 2 kHz. To facilitate identification of bursts of sympathetic nerve activity the filtered signal is also full-wave rectified and displayed as a mean-voltage neurogram, produced as an integrated signal (time constant 100 ms) or as a root mean square (RMS)-processed moving average (time constant 200 ms). The advantage of the moving average is that it does not introduce the 100-ms time lag associated with integration (which uses the “leaky-integrator” resistor–capacitor circuit), and the 200-ms time constant provides a smoother profile of the burst.

**Fig. 2:**
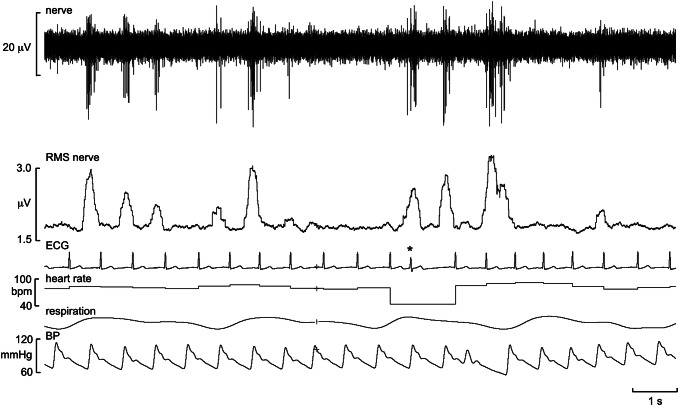
Oligounitary recording of spontaneous bursts of MSNA. Note the low background noise in this recording. An augmented burst follows the ventricular ectopic beat, indicated by the asterisk. Unpublished data from Macefield

**Fig. 3:**
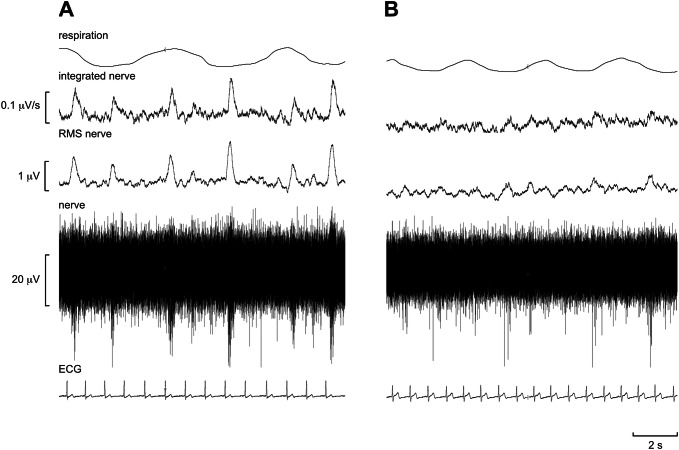
Typical multi-unit (**a**) and oligounitary (**b**) recordings of spontaneous bursts of MSNA. The integrated (100 ms time constant) and RMS-processed (200 ms moving average) mean-voltage neurograms are shown above. Unpublished data from Macefield

An example of a recording of MSNA is shown in Fig. 2. This is an oligounitary recording obtained from a high-impedance microelectrode and is dominated by a few large spikes (oligo = few) generated from axons located close to the microelectrode tip. The RMS-processed signal shows that the mean power in the baseline between bursts is approximately 1.8 μV, and the peak-to-peak background noise is approximately 10 μV. Such low noise levels and high signal–noise ratio indicate a high-quality recording. A low-impedance microelectrode (less than 50 kΩ) will typically record low-amplitude multi-unit activity composed of small spikes generated some distance from the microelectrode tip, so-called far-field activity. Recordings of this type, which are not worth illustrating, can only really be analysed from the mean-voltage neurogram, which allows one to sample the population response despite the raw neurogram being of poor quality, with a signal-to-noise ratio of less than 1.5:1. Conversely, a high-impedance microelectrode is required for making oligounitary and especially unitary recordings. Nevertheless, if the recording site is too focal, and owing to the arrangement of sympathetic axons within the nerve [22], there will be very little far-field activity detected and the mean-voltage neurogram will be small. This can be seen by comparison of the two recordings in Fig. 3. The one on the left is a typical, good-quality multi-unit recording of MSNA: both the integrated and RMS-processed nerve signals show well-defined bursts. Conversely, the one on the right is an oligounitary recording, with very little far-field activity and hence only small fluctuations in the mean-voltage neurogram. One would find counting bursts from either the integrated or RMS-processed signals very difficult in this situation, which is why one should always record the raw nerve signal as well. By examining the latter, one can count the bursts and, as we will see below, obtain quantitative information from this signal.

Note that the action potentials generated by sympathetic axons are negative-going, unlike the positive-going spikes of myelinated axons, such as shown in Fig. 1; these are what one would expect of the voltage transients picked up by a monopolar electrode recording action potentials from the exposed axonal membranes of unmyelinated axons or from the insulated axonal membranes of myelinated axons, in which current fluxes are limited to the nodes of Ranvier. The recording in Fig. 2 also shows an augmented burst of MSNA associated with a long cardiac interval that follows a ventricular ectopic beat. Note also the lag in the generation of this baroreflex-mediated compensatory burst, which largely reflects the time required for the volley of action potentials to arrive at the recording site along slowly conducting unmyelinated axons, as will be discussed below.

**Fig. 4:**
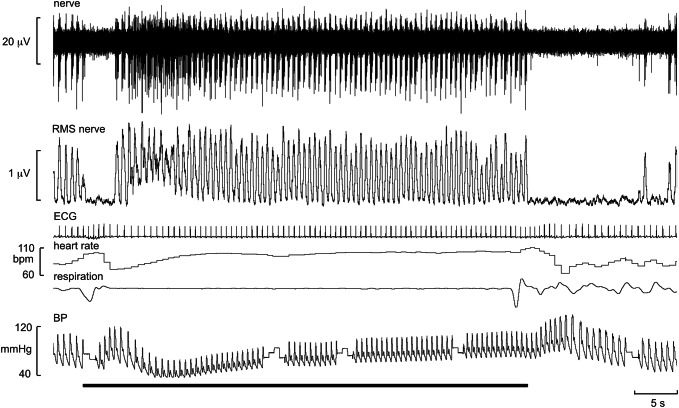
Oligounitary recording of MSNA during an inspiratory-capacity apnoea, indicated by the horizontal bar. MSNA was inhibited during lung inflation and deflation and was markedly increased during the apnoea. The respiration transducer was wrapped around the abdomen in this participant, so the increase in chest expansion during the maximal inspiratory breath-hold is not adequately recorded. Unpublished data from Macefield

Searching for MSNA is facilitated by asking the participant to perform an inspiratory-capacity apnoea (maximal inspiratory breath-hold), an end-expiratory apnoea or a Valsalva manoeuvre, each of which increases MSNA [23]. I prefer the inspiratory-capacity apnoea, which causes a sustained increase in MSNA, after an initial inhibition during the lung-inflation phase, for as long as the breath is held [24]. This is illustrated in Fig. 4. The mode of action here is that holding one’s breath at the end of a maximal inflation is made against a closed glottis, so the only muscles active are the laryngeal constrictors. This differs from the forced expiratory effort of the Valsalva manoeuvre. Nevertheless, both respiratory acts lead to an increase in intrathoracic pressure (as well as intra-abdominal pressure in the case of the Valsalva manoeuvre), due to elastic recoil of the lungs and chest wall in the former and active expiratory effort in the latter, which leads to unloading of the low-pressure baroreceptors and hence withdrawal of the negative feedback and promoting the generation of MSNA [24]. It can be seen that the bursts almost become fused in the early part of the response, due to the marked fall in blood pressure; the sustained increases in MSNA then bring diastolic pressure back towards baseline levels, although pulse pressure remains low. Following the exhalation there is inhibition of MSNA as blood pressure overshoots; one should wait for spontaneous bursts to reappear before conducting further manoeuvres.

**Fig. 5:**
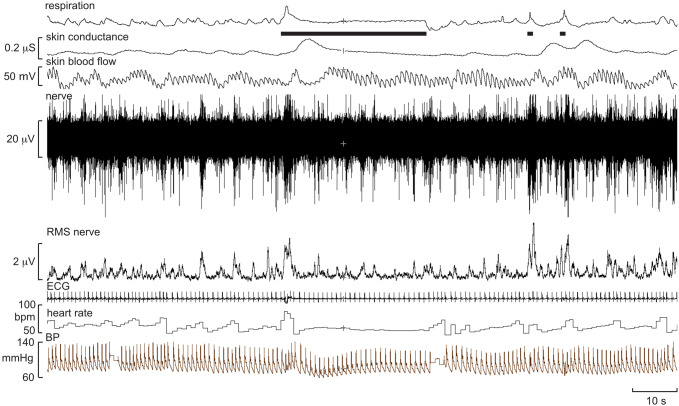
Multi-unit recording of skin sympathetic nerve activity (SSNA) during an inspiratory-capacity apnoea, indicated by the long horizontal bar, and during two brisk sniffs, indicated by the short bars. Skin conductance, indicating sweat release, was recorded between two toes. A proxy marker of skin blood flow was obtained from the pulsatile changes in perfusion, measured by a photoelectric sensor on the pad of a toe. Brief bursts of SSNA were evoked during lung inflation and deflation, but there was no sustained increase during the inspiratory-capacity apnoea. Unpublished data from Macefield

One defines the innervation territory of a cutaneous fascicle by light stroking of the skin, which—as long as the stimuli are not sufficient to induce muscle stretch—does not generate afferent responses when recording from a muscle fascicle. Occasionally, one may encounter spontaneous bursts of SSNA, particularly if the participant is anxious, but—like MSNA—you generally need to search for it. The respiratory manoeuvres mentioned above do not increase SSNA, which is less affected by baroreflexes [25, 26]. Unexpected stimuli, such as a shout, tap on the head, or brisk sniffs evoke single bursts of SSNA, and a subsequent increase in sweat release and vasoconstriction (Fig. 4). Bursts of SSNA are longer than those of MSNA, the latter being shorter because their bursts are terminated by the arterial baroreflex during systole; when the baroreceptors are blocked, MSNA takes on much of the character of SSNA [27]. As shown in Fig. 5, the inspiratory-capacity apnoea causes a burst of SSNA during the inflation and deflation phases, but no sustained increase during the apnoea; here the activity during the breath-hold is the same as at baseline, which was fairly high in this individual. Given that SSNA is composed of the activity of sudomotor neurons supplying sweat glands, cutaneous vasoconstrictor neurons supplying blood vessels, as well as—in hairy skin—pilomotor neurons supplying the hairs, SSNA can be high both when the participant is hot or cold. In thermoneutral conditions SSNA is fairly low, but this activity—which is dominated by cutaneous vasoconstrictor activity—will be elevated when the participant is anxious, as in the example shown in Fig. 5. This is particularly so when recording SSNA from cutaneous fascicles supplying the glabrous (non-hairy) skin, such as that on the volar aspect of the hand or the plantar aspect of the foot. It is known that heat transfer by the glabrous skin of the hand is determined primarily by skin blood flow, as determined by cutaneous vasoconstrictor neurons, rather than by the activity of sudomotor neurons [28–30]. Indeed, heat-induced activation of sudomotor neurons supplying the hand only occurs at ambient temperatures higher than 45 ℃, and that—in the absence of an arousal stimulus (which produces coactivation of cutaneous vasoconstrictor and sudomotor neurons)—any ongoing SSNA recorded from the median nerve at the wrist largely reflects cutaneous vasoconstrictor drive [28, 29]. Moreover, it follows that, in thermoneutral conditions, spontaneous bursts of SSNA directed to the glabrous skin of the hand are governed primarily by emotion, rather than thermoregulation. This is particularly so in the case of idiopathic palmar hyperhidrosis, in which spontaneous bursts of SSNA reflect an increase in firing probability and recruitment of sudomotor neurons [31]. The same is true for the foot: individuals with plantar hyperhidrosis have elevated SSNA to the sole of the foot (as recorded from the tibial nerve) during emotional arousal, whereas SSNA to the hairy skin (as recorded from the common peroneal nerve) in these individuals is no different at rest or during mental and thermal stimuli [32].

**Fig. 6:**
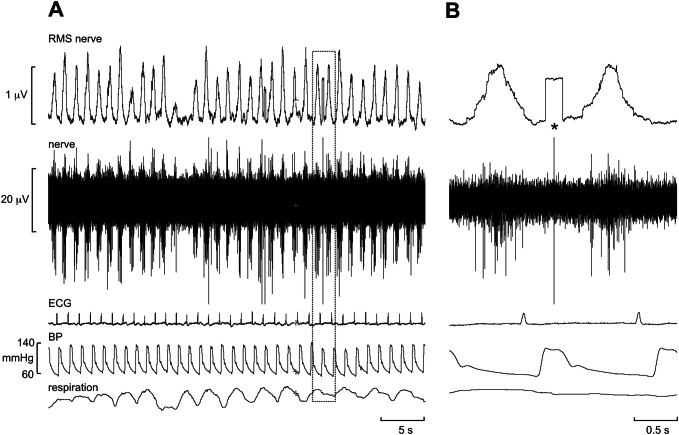
**a** Multi-unit recording of spontaneous bursts of MSNA in a patient with renovascular hypertension. Note that bursts occur in most cardiac intervals. **b** Expanded recording of the section outlined in** a**. Note that the brief electrical artifact, indicated by the asterisk, causes a characteristic square-wave profile in the RMS-processed mean-voltage neurogram. Unpublished data from Macefield

It is important to recognise that levels of both MSNA and SSNA differ across individuals at rest, with the levels of MSNA being similar in identical (but not fraternal) twins and consistent over months and years [33, 34]. Moreover, resting levels of MSNA in healthy individuals bear no relationship to resting blood pressure or heart rate [35]. However, given that sympathoexcitation is a feature of many cardiovascular and respiratory diseases it is not surprising that there are higher levels of baseline MSNA in individuals with neurogenic hypertension, heart failure, kidney failure, obstructive sleep apnoea and many other conditions—which makes identification of spontaneous bursts easier. An example of a multi-unit recording of MSNA from a patient with renovascular hypertension, in which bursts occur in most cardiac intervals, is shown in Fig. 6. However, high levels of MSNA at rest do not necessarily imply pathophysiology: some young healthy individuals do have high resting MSNA and normal blood pressure [33, 36], and the firing properties of individual muscle vasoconstrictor neurons recorded in such individuals do not reveal the different patterns of firing seen in individuals with cardiovascular disease [37]. MSNA is higher in men than women, and MSNA is positively correlated to total peripheral resistance in young men but not women, in whom β-adrenergic vasodilator mechanisms counteract α-adrenergic vasoconstriction, and in both groups MSNA increases with age [36, 38, 39]. Moreover, the relationship between MSNA and blood pressure becomes tighter after the age of 40 [35, 37, 38], and the age-related increase in MSNA has been shown to contribute to the arterial stiffening seen in older adults [40]. Another factor affecting resting MSNA is body weight: MSNA is higher, and baroreflex function blunted, in overweight and obese individuals [41, 42]. Finally, when recording MSNA, the overall levels will be determined by body position: it is generally low in the supine position, because of the lack of gravitational unloading of the low-pressure baroreceptors, and higher in the seated position. Because of this, I routinely record MSNA with the participant in a semi-recumbent position; this also allows the participant to see the computer monitor and be actively engaged in the recording session.

## Noise, artifacts and stability

Electrical noise is the bane of a neurophysiologist’s life, and it is worth considering how to reduce this. Amplifiers for microneurography are, like those for other neurophysiological applications, differential amplifiers. In other words, they measure the potential difference (voltage) between one electrode (the active electrode) and another (the reference electrode). In practice, for our purposes the active electrode is a high-impedance tungsten microelectrode inserted into a fascicle of a nerve, while the reference is an uninsulated tungsten microelectrode inserted into the skin approximately 1 cm away. To complete the picture, one needs an electrode that gets rid of everything else—the so-called common mode. This is the ground electrode, and we typically will use a disposable Ag/AgCl surface electrode for this, which can be placed anywhere on the body (but in practice is located close to the headstage of the amplifier). Modern amplifiers have a high input impedance, meaning the voltage picked up by the recording (active) microelectrode is maintained as high as possible, without current leakage into the amplifier. Because the input impedance is high, and the electrode impedance is high, anything between the tip of the microelectrode (the recording surface devoid of insulation) and the input terminals of the headstage—the connecting wires—will act as antennae that pick up electrical noise, as noted above. And while some laboratories will set up expensive shielded rooms to protect them from radio frequency (RF) interference, which sits in the bandwidth of axonal action potentials, the greatest sources of noise (the line frequency of the power supply, 50 or 60 Hz) sit within the laboratory (i.e. the data acquisition and monitoring equipment), as do the sources of higher frequency components, such as computer monitors and fluorescent lights. There is a limit to how much we can reduce the noise, but certainly having a high pass filter of 100 Hz or above helps, as does having a headstage that preamplifies the signal at least by 10. For example, the NeuroAmpEX amplifier and headstage by ADInstruments, for which I served as the design consultant (providing the specifications and testing all protoypes), has software-controlled analog and digital amplifiers, with the headstage having a bandpass of 100 Hz to 5 kHz and a gain of ×100. All of the recordings I have illustrated were obtained using the NeuroAmpEX amplifier and recorded on LabChart (ADInstruments, Australia). Some amplifiers I have used in the past have a headstage gain of 1! Needless to say, a headstage with unity gain will mean that the high-impedance microelectrode and connecting wires will pick up a lot of electrical interference, which can be reduced by wrapping aluminium foil around the recording site, with conductive gel between the foil and the participant’s skin. The background noise for a typical high-impedance (approximately 1–5 MΩ) microelectrode will be approximately 20 μV peak-to-peak. When the microelectrode tip loses some of its insulation, through repeated passage through fascicles or the nerve sheath or skin (one should avoid removing the microelectrode completely and reinserting through the skin), the background noise becomes appreciably smaller and the recording capacity of the microelectrode will be compromised: at impedances typically less than 100 kΩ, the microelectrode will only pick up far-field nerve activity.

Another aspect we need to consider is stability. We are, of course, at the mercy of the participants here, given that we are asking them to keep as still as possible. While stable recordings of sympathetic nerve activity can be obtained for well over an hour, one needs to be aware of potential changes in the recording site over time. If the nerve is quite deep and the microelectrode well supported by the tissues overlying the nerve, the microelectrode should remain in a stable intrafascicular site for a long time. Conversely, when the nerve is superficial the microelectrode, particularly if directed towards the nerve in an upward trajectory, may tend to slip out through the effects of gravity. This will be seen as a downward shift in the baseline of the mean-voltage neurogram, which may be progressive or occur abruptly. This makes it difficult to compare bursts of sympathetic nerve activity recorded before and after this shift, particularly as the burst amplitudes are likely to be much smaller following a site change and what was previously a small but measurable burst is now too small to detect, thereby leading to a difference in the calculated burst frequency and burst incidence. In addition to baseline shifts, one needs to be aware of the effects of an electrical artifact on the integrated and RMS-processed signals: such a brief electrical spike causes a characteristic shape in the mean-voltage neurogram that can easily be recognized, as seen in Fig. 6.

## Analysis of sympathetic nerve activity from the mean-voltage neurogram

**Fig. 7:**
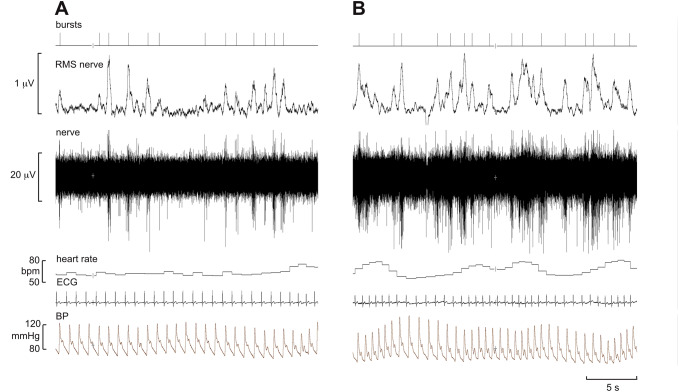
Multi-unit recordings of spontaneous bursts of MSNA (**a**) and SSNA (**b**) in two participants. The Cyclic Measurements feature of LabChart was used to automatically identify bursts, based on template matching, as indicated by event markers in the top trace. Unpublished data from Macefield

It has been known since the first direct recordings of sympathetic neural traffic were made in humans that sympathetic outflow occurs not as a continuous (tonic) discharge but as bursts of impulses [23, 25], and this is why the term “sympathetic tone” can be misleading. Because the unmyelinated postganglionic axons tend to travel together within a fascicle [22], a typical recording is composed of action potentials generated by many axons. A multi-unit recording is most often analysed simply by counting the number of bursts in 1 min (burst frequency) or in 100 heart beats (burst incidence), usually from the mean-voltage neurogram (the integrated nerve signal or RMS-processed signal). The advantage of burst incidence is that it takes into account differences in heart rate across individuals, but in practice both measures are usually used. Nevertheless, these are very coarse metrics and depend on the threshold one applies to accepting an increase in mean-voltage neurogram as being a real sympathetic burst or not. Indeed, it is for this reason that visual counting of bursts in a given study needs to be performed by the same investigator. To make detection less dependent on the operator, there are several automated programs available for analysis of sympathetic nerve activity, in particular MSNA, which look for a burst within a certain time window following an R wave of the electrocardiogram (ECG). This is based on the principle that bursts of MSNA are tightly coupled to the ECG through the arterial baroreflex, and for bursts of MSNA recorded from the common peroneal nerve these occur approximately 1.2–1.3 s after the R wave (though 100 ms shorter if one uses a moving-average RMS signal, which introduces no time lag), the absolute latency being primarily determined by the length of the slowly conducting unmyelinated axons in the limbs (the latency is longer in taller people). Such programs also measure the amplitude of a burst, which is an important metric when considering the effects of a manoeuvre on sympathetic outflow. Because the amplitude (and area) of a burst is determined by the number of neurons active, the proximity of the microelectrode tip to those axons, as well as the size of the action potentials generated by those axons (larger axons generate larger spikes), one cannot compare amplitudes across individuals or studies, only within a given recording session. In this case, all bursts are normalized to the largest burst recorded within that session. It is then possible to compare the distribution of normalized burst amplitudes: patients with heart failure, for example, will show a distribution of burst amplitudes that are skewed towards the right, i.e. to larger bursts than the normal distribution seen in healthy individuals [43–45]. Other approaches use template matching to identify peaks in the mean-voltage neurogram, two examples of which are shown in Fig. 7. This approach uses the standard deviation of the integrated or RMS-processed neurogram for thresholding, the operator setting the level at which a peak is accepted as a burst. This allows bursts to be detected and their amplitudes measured automatically, and works for detection of bursts of both MSNA and SSNA. Nevertheless, all automatic or quasi-automatic burst analysis methods do require operator oversight to ensure that artifacts have not been counted as bursts (false positives) or that smaller bursts have not been missed (false negatives).

**Fig. 8:**
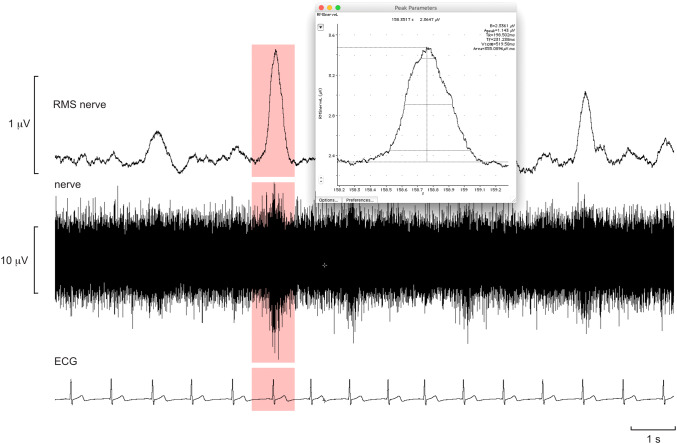
Multi-unit recording of spontaneous bursts of MSNA. The neurograms have been shifted back in time 1.2 s to align the bursts with the relevant R wave of the ECG, and the 1-s period around this peak used to measure bursts. The inset shows the highlighted burst in the Peak Parameters feature in LabChart, which allows one to extract various aspects of the burst, including the baseline over the first 100 ms (B), peak amplitude (*A*_peak_), rise time (*T*_R_), fall time (*T*_F_), burst duration (*W*_10%_) and area. Unpublished data from Macefield

Rather than measuring bursts through threshold crossing or template matching one can simply measure the mean-voltage neurogram in every cardiac interval. This works best for MSNA because each burst is constrained by the arterial baroreflex, the peak of the burst reflecting the onset of its termination by the baroreflex. One still needs to take into account conduction delays, as pointed out above, so in practice the nerve signal can be shifted back in time by approximately 1.2–1.3 s such that the burst now appears in the cardiac interval responsible for its generation. Then one can write a simple routine that will jump from R wave to R wave and measure the mean-voltage neurogram, or preferably the features of a burst (amplitude, area, duration, rise time, etc.) in the 1-s epoch that straddles that R wave, together with other relevant parameters such as the duration of the cardiac interval, systolic and diastolic pressure, etc. The selection approach is shown in Fig. 8. The advantage of this method is that it quickly analyses an entire file, or part thereof, unsupervised. Each row in the text file so generated corresponds to a single cardiac interval, sampled consecutively, and the columns of measured parameters are obtained between that R wave and the next. This can generate a lot of data, and then it is simply up to the operator to determine the minimal amplitude of a peak that will be classified as a burst: those below these are termed non-bursts, so by knowing the total number of R waves in the recording (or extract) one can easily obtain the proportion of bursts and non-bursts and thereby calculate burst frequency, burst incidence, cumulative (total) burst amplitude and burst amplitude distribution. And, because diastolic pressure has also been measured, together with any other variable of interest, one can compute the relationship between burst amplitude and diastolic pressure and calculate baroreflex sensitivity and gain. While sympathetic outflow to skin does not exhibit overt cardiac rhythmicity, it is actually present. As detailed below, the cardiac modulation of SSNA can be quantified [46, 47], but is much weaker than that of MSNA. Nevertheless, this means that similar approaches can be used to analyse SSNA, with the caveat that the bursts are broader and more variable in shape.

## Analysis of sympathetic nerve activity from the filtered neurogram

**Fig. 9:**
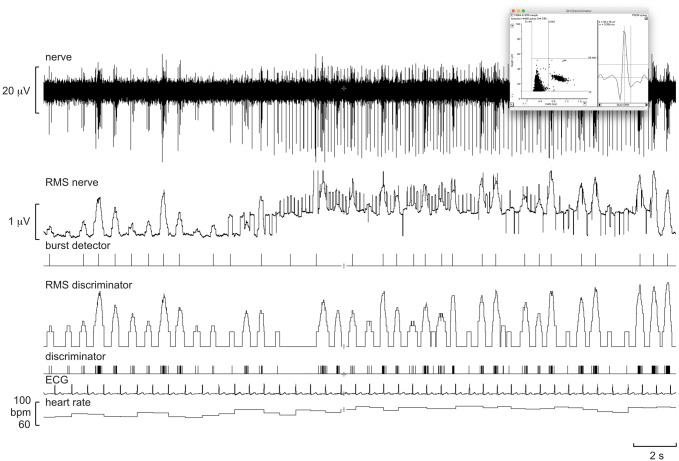
Oligounitary recording of MSNA in which EMG activity infiltrated the signal. The inset shows the Spike Histogram feature of LabChart, in which the narrow sympathetic spikes can be separated from the wider EMG spikes, and the bursts reconstructed from these discriminated spikes. Unpublished data from Macefield

While the above means of quantifying sympathetic nerve activity are useful, the information content of the integrated or RMS-processed nerve signal is still fairly low. Other approaches are based on an analysis of the raw nerve signal, filtered to remove obvious signs of electrical interference. Counting action potentials is a well-established method of quantifying a nerve signal, having been used for decades in neurophysiological research involving experimental animals. The principle here is to use a time–amplitude window discriminator (originally hardware, but now mostly software-based) to extract the spikes of interest. I and others have used this to quantify the firing properties of single postganglionic sympathetic neurons, which I shall not consider further here as it has been reviewed recently [7]. Of course, this approach can also be used to quantify a multi-unit or oligounitary recording, the difference being that one counts the spikes originating from many active axons, not just one. Importantly, as noted above, we know that postganglionic sympathetic axons generate negative-going spikes, making it easier to extract these from the positive-going spikes of myelinated axons, such as muscle spindle afferents, and from the wider action potentials generated by motor units. This is shown in Fig. 9, in which some electromyographic (EMG) activity infiltrated the recording as the participant tensed her leg. The inset shows the spike discriminator, with spike-like events being plotted as a function of amplitude (vertical axis) and width (horizontal axis); the narrow neural spikes cluster to the left, whereas the wider EMG potentials are clustered to the right. One of the selected negative-going sympathetic spikes is also shown in the discriminator window, inverted (the discriminator only operates on positive spikes, so the signal is inverted to capture the sympathetic spikes). These discriminated spikes are shown in the discriminator output trace in the lower part of the figure. It can be seen that one can construct an RMS-processed signal from these extracted spikes which, given that the EMG interference has been removed, now allows one to measure the amplitude and area of this reconstructed signal of MSNA. It can also be seen that the automatic method of detecting bursts on the basis of a template (see the “bursts” trace in the figure) performs quite well at detecting bursts despite the EMG interference, but it still makes measuring burst amplitude and area difficult because of the upward shift of the baseline.

**Fig. 10:**
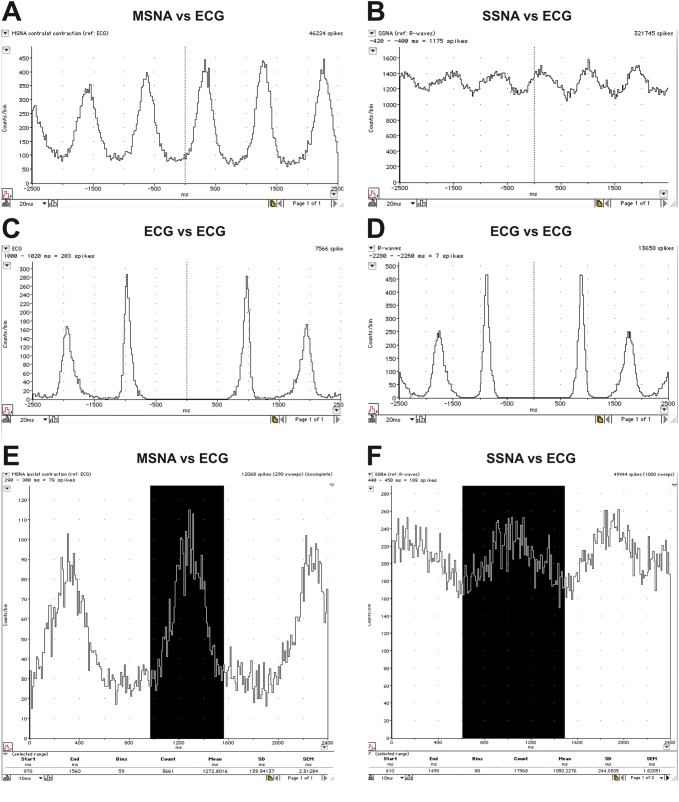
Cross-correlation histograms between MSNA and the ECG (**a**) and SSNA and the ECG (**b**). Sympathetic spikes were extracted from the neurogram using Spike Histogram feature, shown in Fig. 9. The corresponding autocorrelation histograms for the ECG are shown in **c** and **d**. Post-stimulus time histograms of the same data, from which the total number of sympathetic spikes generated during the average burst can be counted, are shown in **e** and **f**. Unpublished data from Macefield

Once the spike events have been extracted, one can also construct cross-correlation histograms (correlograms) between MSNA or SSNA and the ECG. By plotting the times of occurrence of each spike as a function of the times of occurrence of each R wave of the ECG, one can document and quantify the cardiac modulation of both muscle and skin sympathetic nerve activity. Examples are shown in Fig. 10a, b, together with the corresponding auto-correlograms of the ECG in Fig. 10c, d. It is immediately obvious that the cardiac modulation of MSNA is stronger than that of SSNA. This is not surprising, given that muscle vasoconstrictor neurons are strongly entrained to the cardiac rhythm via the arterial baroreflex, but one needs to appreciate the fact that individual sudomotor and cutaneous vasoconstrictor neurons also exhibit cardiac modulation [48, 49], meaning that multi-unit recordings of SSNA are also modulated by the cardiac rhythm, although this is much weaker than that of MSNA [46, 47]. Another method, using the same data, allows one to quantify the mean burst intensity in terms of the total number of spikes generated in an averaged burst, calculated by constructing the equivalent of a post-stimulus time histogram (PSTH) by using the R waves as the “stimulus”. So, for a given number of R waves (say, 100), one can determine the total number of spikes in this averaged burst, which includes cardiac intervals in which there are no bursts. As shown in Fig. 10e, f the highlighted area shows the selected bins comprising the averaged burst tied to the R wave at time zero (the “stimulus” event). This approach avoids setting a threshold for accepting a peak in the mean-voltage neurogram as a burst, but it does of course depend on setting a threshold for accepting spikes as originating from sympathetic axons. Nevertheless, this mean burst intensity is only useful when comparing manoeuvres in a given recording; it still cannot be used to compare recordings across individuals and across studies, as the number of sympathetic spikes in the recordings will differ.

Another means of extracting more information from the raw neural signal is based on wavelet transform analysis, which uses a spike template (wavelet) to extract nerve spikes of differing shape and amplitude and assign them to clusters. This was developed by André Diedrich and colleagues [49, 50] and explored further by Kevin Shoemaker’s group to assess how a population of muscle vasoconstrictor neurons behaves at rest and during physiological manoeuvres that increase MSNA [51, 52]. This approach, the subject of an excellent recent review [8], has complemented the single-unit approach, which showed that (1) individual postganglionic sympathetic axons primarily fire only once in a sympathetic burst and that (2) increases in burst intensity are brought about by an increase in firing probability of active neurons and, by inference, (3) an increase in the recruitment of neurons [7]. While the wavelet approach cannot provide information on the firing rates of individual neurons it has provided direct evidence that recruitment of previously silent neurons plays a dominant role in physiological increases in sympathetic nerve activity. Most recently, it has also uncovered evidence that not all muscle vasoconstrictor neurons fire in a burst-like fashion: some fire asynchronously, i.e. in the intervals between bursts of MSNA [53]. Moreover, recent single-unit recordings have shown that not all muscle vasoconstrictor neurons behave in the expected fashion during physiological increases in MSNA: some behave paradoxically, their firing probability and firing rates decreasing rather than increasing [54].

## Conclusions

As recently reviewed by Carter [4], direct microelectrode recordings of sympathetic outflow in awake humans have contributed a great deal to our understanding of the physiology and pathophysiology of the sympathetic nervous system. Sympathetic microneurography is undertaken in many laboratories throughout the world, and there are many differences in how the recordings are made and analysed. My hope in providing this review is to give new students a greater insight into what they will be contributing to the field, as there is still so much more to learn. While I trust I have covered the methods and caveats, the challenges are limited only by one’s imagination. Indeed, it was as a result of our desire to understand where in the brain sympathetic outflow to muscle and skin is generated that we tried and succeeded in developing the methodology for recording sympathetic nerve activity at the same time as performing functional magnetic resonance imaging (fMRI) of the brain: MSNA-coupled fMRI and SSNA-coupled fMRI [55–57].
